# Supplementary material for: Wild Patagonian yeast improve the evolutionary potential of novel interspecific hybrid strains for lager brewing
Source: PLoS Genet. 2024 Jun 20;20(6):e1011154. doi: 10.1371/journal.pgen.1011154 (PMC11189258; doi:10.1371/journal.pgen.1011154)
Supplement: S1 Fig — Experimental procedure designed to generate and identify interspecific hybrids at two different temperatures (12 and 20°C). (PDF) [file pgen.1011154.s001.pdf]

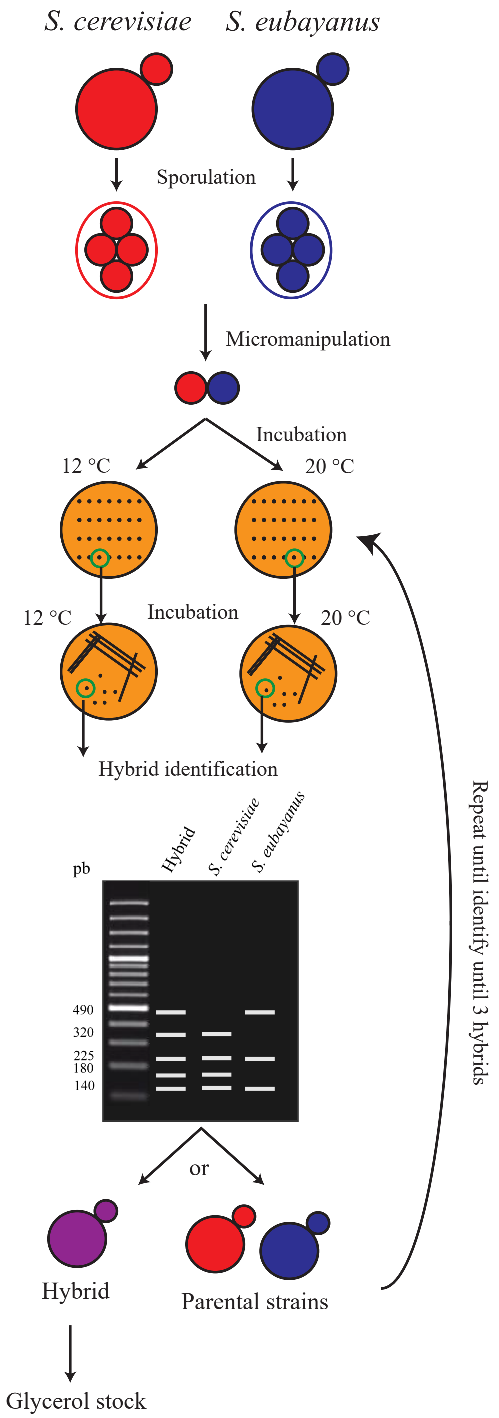

Figure S1. Generation of interspecific *S. cerevisiae* x *S. eubayanus* hybrids. Experimental procedure designed to generate and identify interspecific hybrids at two different temperatures (12 and 20°C).
