# Supplementary material for: Wild Patagonian yeast improve the evolutionary potential of novel interspecific hybrid strains for lager brewing
Source: PLoS Genet. 2024 Jun 20;20(6):e1011154. doi: 10.1371/journal.pgen.1011154 (PMC11189258; doi:10.1371/journal.pgen.1011154)
Supplement: S2 Fig — (A) Fermentation performance of 15 S. cerevisiae strains. (B) Maximum OD reached of growth curves in maltotriose 2% under microculture conditions (C) Maltotriose uptake after growth in maltotriose 2% under microculture conditions. Plotted values correspond to three biological replicates. The (*) represents different levels of significance between the phenotype of haploid strains and their respective parental strain (t-test; *p ≤ 0.05, **p≤ 0.01, ***p≤ 0.001, ****p≤ 0.0001 and ns: non-significant). (PDF) [file pgen.1011154.s002.pdf]

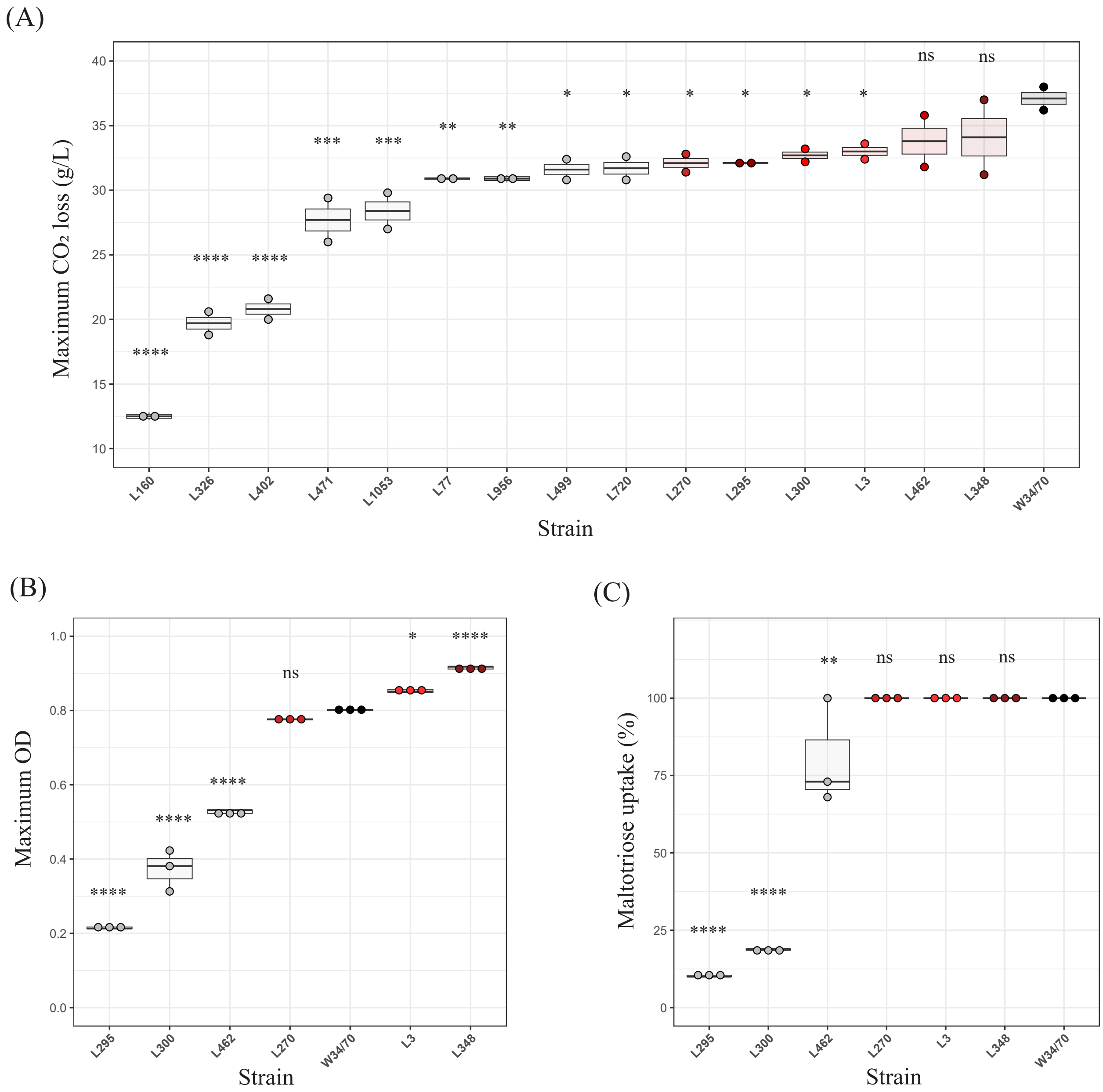

Figure S2. Phenotypic characterization of *S. cerevisiae* parental strains. (A) Fermentation performance of 15 *S. cerevisiae* strains. **(B)** Maximum OD reached of growth curves in maltotriose 2% under microculture conditions **(C)** Maltotriose uptake after growth in maltotriose 2% under microculture conditions. Plotted values correspond to three biological replicates. The (*) represents different levels of significance between the phenotype of haploid strains and their respective parental strain (t-test; *p ≤ 0.05, **p≤ 0.01, ***p≤ 0.001, ****p≤ 0.0001 and ns: non-significant)
