# Supplementary material for: Wild Patagonian yeast improve the evolutionary potential of novel interspecific hybrid strains for lager brewing
Source: PLoS Genet. 2024 Jun 20;20(6):e1011154. doi: 10.1371/journal.pgen.1011154 (PMC11189258; doi:10.1371/journal.pgen.1011154)
Supplement: S3 Fig — Each plot represents a different cross. The (*) represents different levels of significance between the phenotype of hybrids and their respective parental strain (t-test; *p ≤ 0.05, **p≤ 0.01, ***p≤ 0.001, ****p≤ 0.0001). (PDF) [file pgen.1011154.s003.pdf]

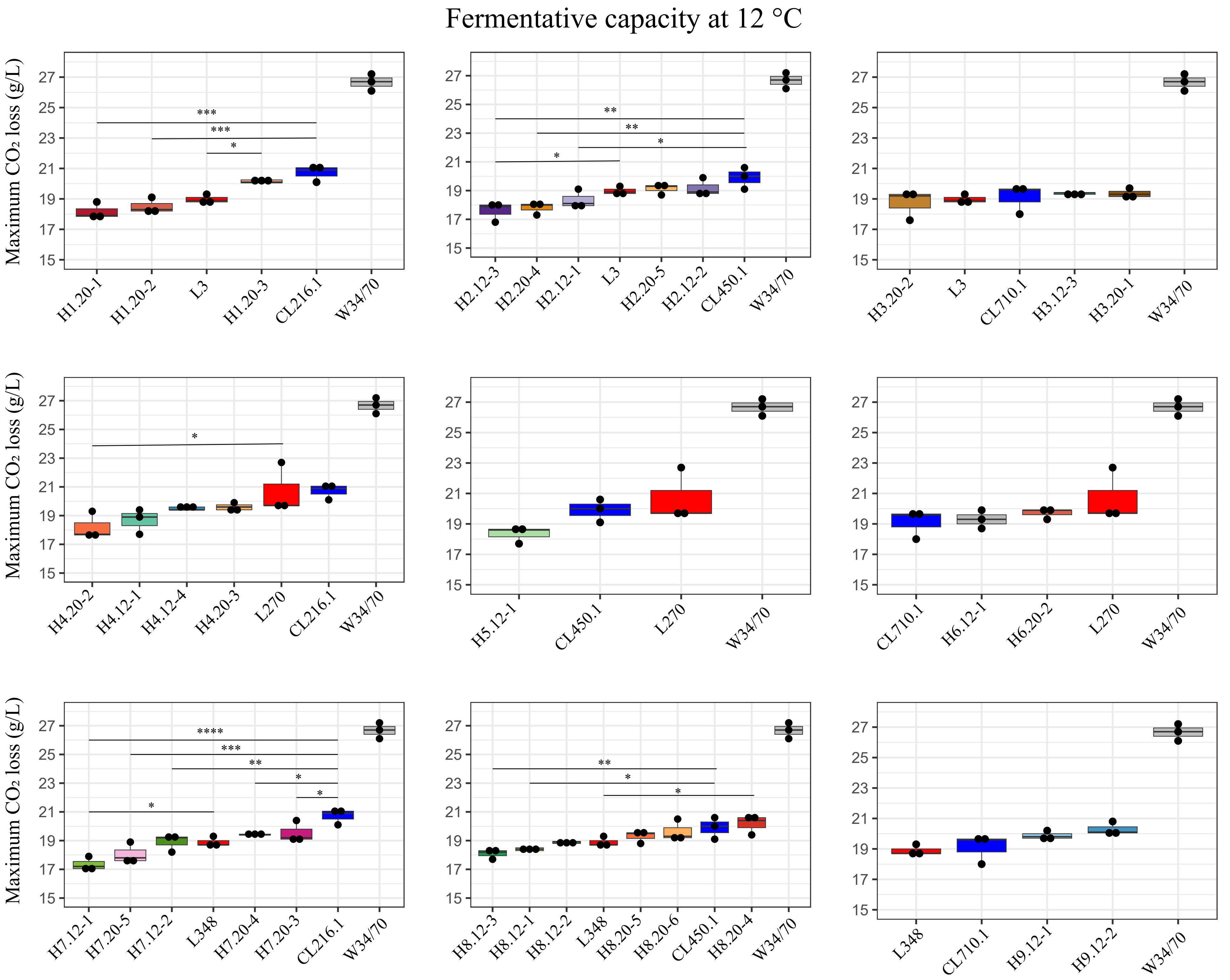

Figure S3. Fermentative capacity at 12 °C of each hybrid. Each plot represent a different cross. The (*) represents different levels of significance between the phenotype of hybrids and their respective parental strain (t-test; *p ≤ 0.05, **p≤ 0.01, ***p≤ 0.001, ****p≤ 0.0001).
