# Supplementary material for: Wild Patagonian yeast improve the evolutionary potential of novel interspecific hybrid strains for lager brewing
Source: PLoS Genet. 2024 Jun 20;20(6):e1011154. doi: 10.1371/journal.pgen.1011154 (PMC11189258; doi:10.1371/journal.pgen.1011154)
Supplement: S4 Fig — (A) Mean relative fitness (growth rate) of evolved lines after 250 generation under microculture conditions. Evolved lines were evaluated in the same medium where they were evolved (M o T medium). (B) Mean relative fitness (growth rate) comparison between evolved lines from hybrids generated at 12 and 20°C. Plotted values correspond to the mean of three independent replicates of each evolved lines. The (*) represents different levels of significance between evolved lines and unevolved hybrid in (A) and from hybrids generated at 12°C vs 20°C in (B) (Students t-test, * p < 0.05, ** p < 0.01, ns not significant). (PDF) [file pgen.1011154.s004.pdf]

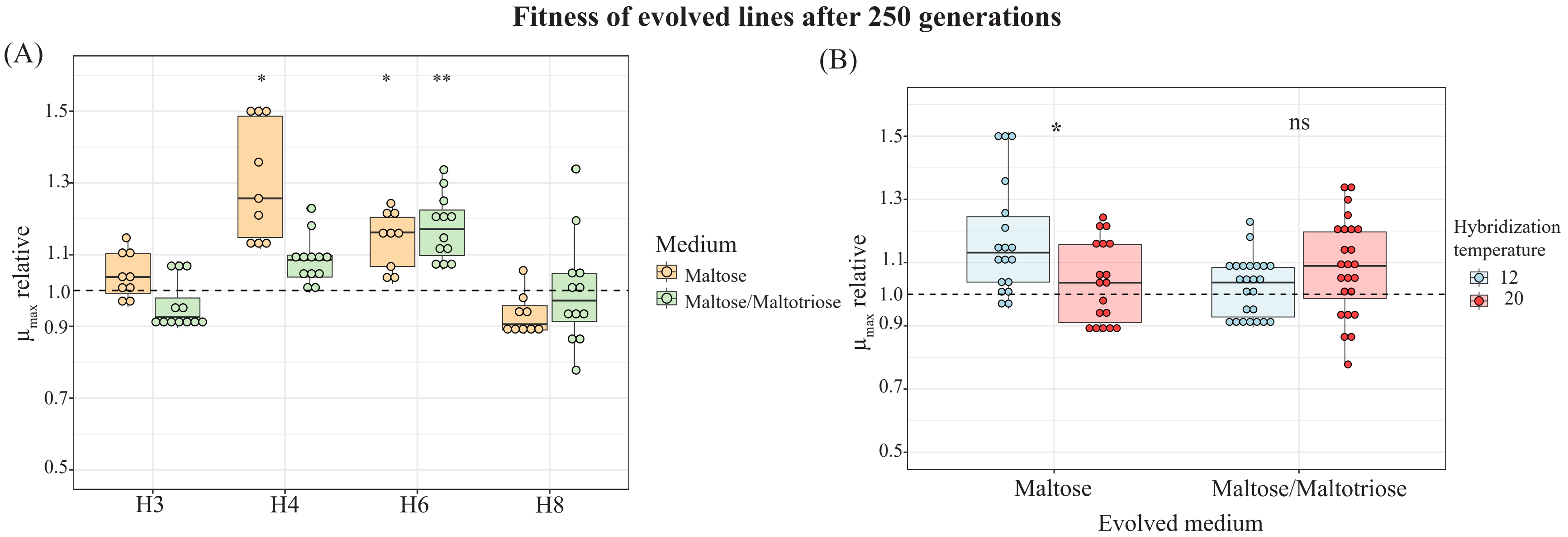

Figure S4. Fitness comparison of evolved lines after 250 generations. (A) Mean relative fitness (growth rate) of evolved lines after 250 generation under microculture conditions. Evolved lines were evaluated in the same medium where they were evolved (M or T medium). **(B)** Mean relative fitness (growth rate) comparison between evolved lines from hybrids generated at 12 and 20 °C. Plotted values correspond to the mean of three independent replicates of each evolved lines. The (*) represents different levels of significance between evolved lines and unevolved hybrid in (A) and from hybrids generated at 12 °C vs 20 °C in (B) (Students t-test, * $p < 0.05$, ** $p < 0.01$, ns not significant).
