# Supplementary material for: Wild Patagonian yeast improve the evolutionary potential of novel interspecific hybrid strains for lager brewing
Source: PLoS Genet. 2024 Jun 20;20(6):e1011154. doi: 10.1371/journal.pgen.1011154 (PMC11189258; doi:10.1371/journal.pgen.1011154)
Supplement: S5 Fig — (A) Mean relative fitness (growth rate and OD) of replicate population in 2% maltose. (B) Mean relative fitness (growth rate and OD) of replicate population in 1% maltose and 1% maltotriose. Plotted values correspond to the mean of three independent replicates of each evolved line. (PDF) [file pgen.1011154.s005.pdf]

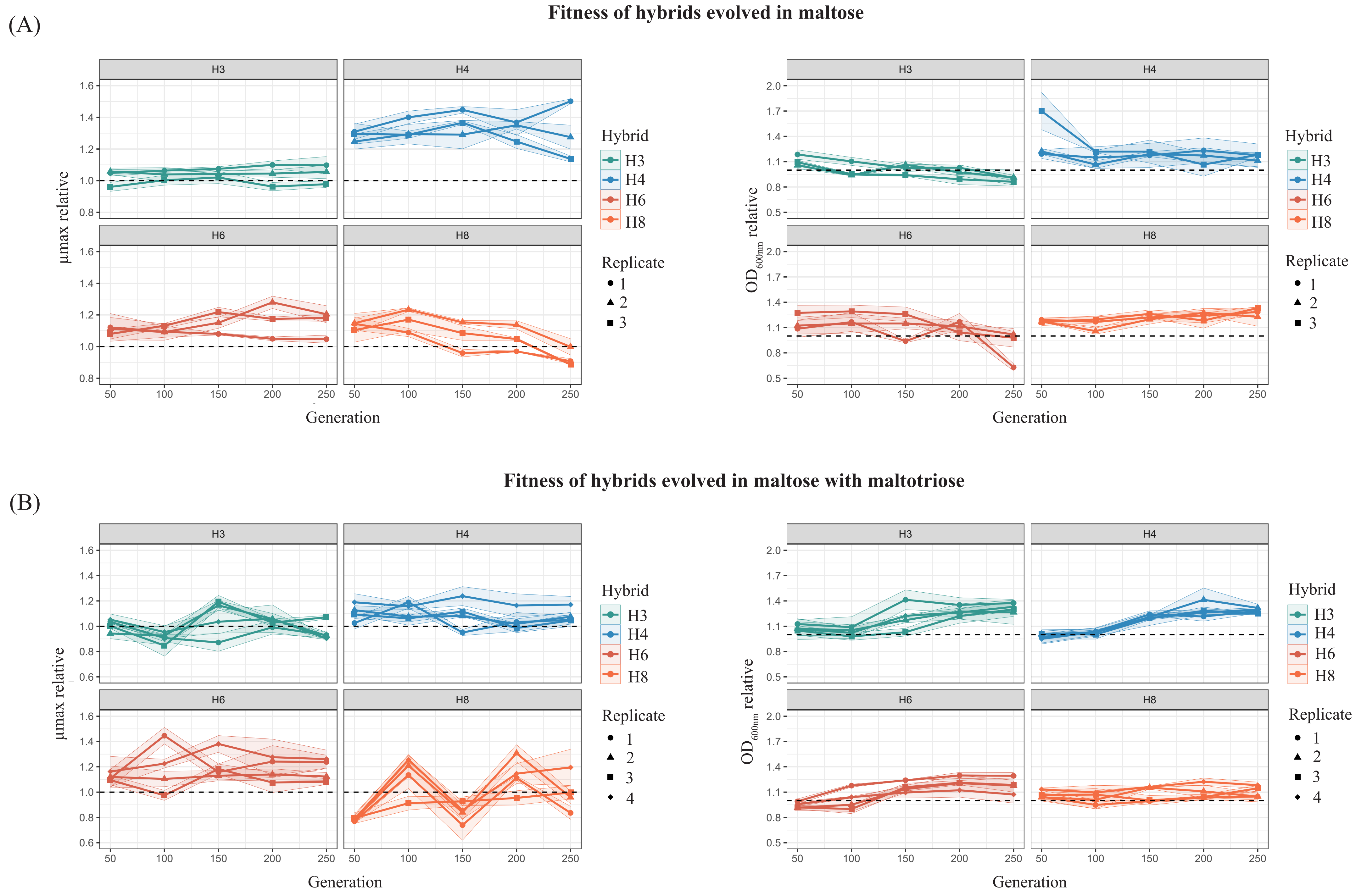

Figure S5. Fitness dynamics of evolved lines in maltose and maltose with maltotriose. (A) Mean relative fitness (growth rate and OD) of replicate population in 2% maltose. (B) Mean relative fitness (growth rate and OD) of replicate population in 1% maltose and 1% maltotriose. Plotted values correspond to the mean of three independent replicates of each evolved line. All values are relative to their respective ancestral hybrid.
