# Supplementary material for: Wild Patagonian yeast improve the evolutionary potential of novel interspecific hybrid strains for lager brewing
Source: PLoS Genet. 2024 Jun 20;20(6):e1011154. doi: 10.1371/journal.pgen.1011154 (PMC11189258; doi:10.1371/journal.pgen.1011154)
Supplement: S6 Fig — (A) Mean relative fitness (maximum CO2 loss) of replicate population in 2% maltose. (B) Mean relative fitness (maximum CO2 loss) of replicate population in 1% maltose and 1% maltotriose. Plotted values correspond to the mean of three independent replicates of each evolved line. (PDF) [file pgen.1011154.s006.pdf]

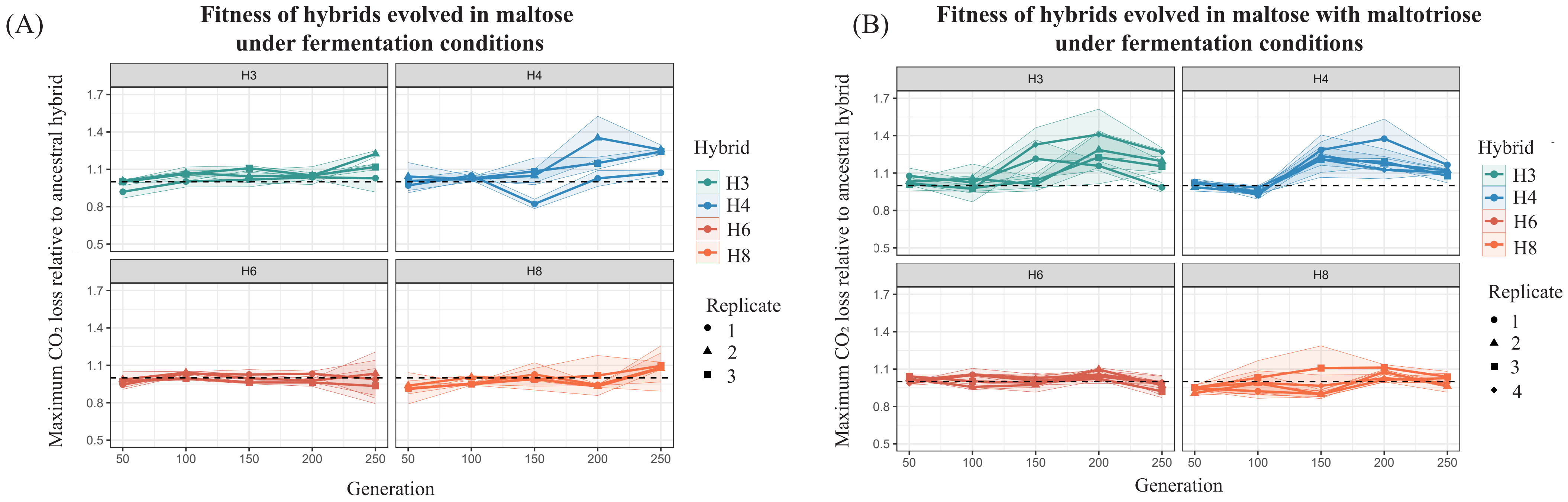


**Figure S6. Fitness dynamics of evolved lines in maltose and maltose with maltotriose under fermentation condition.**
(A) Mean relative fitness (maximum $CO_2$ loss) of replicate population in 2% maltose. (B) Mean relative fitness (maximum $CO_2$ loss) of replicate population in 1% maltose and 1% maltotriose. Plotted values correspond to the mean of three independent replicates of each evolved line.
