# Supplementary material for: Wild Patagonian yeast improve the evolutionary potential of novel interspecific hybrid strains for lager brewing
Source: PLoS Genet. 2024 Jun 20;20(6):e1011154. doi: 10.1371/journal.pgen.1011154 (PMC11189258; doi:10.1371/journal.pgen.1011154)
Supplement: S7 Fig — Fermentative capacity of evolved individuals relative to the commercial lager strain W34/70. Plotted values correspond to the mean of three independent replicates of each individual. The (*) represents different levels of significance between strains and commercial lager strain (Students t-test, * p < 0.05, ** p < 0.01, *** p < 0.001). (PDF) [file pgen.1011154.s007.pdf]

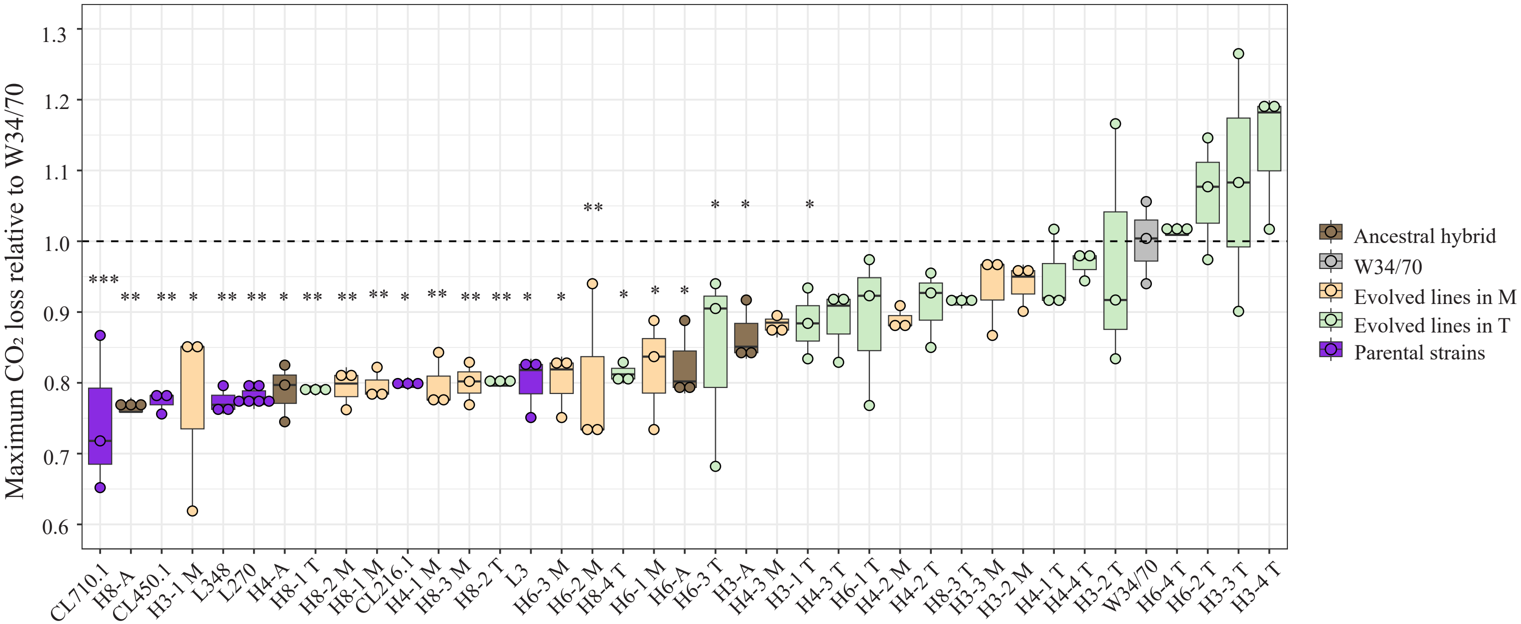

Figure S7. Fermentative capacity of evolved individuals.

Fermentative capacity of evolved individuals relative to the commercial lager strain W34/70. Plotted values correspond to the mean of three independent replicates of each individual. The (*) represent different levels of significance between strains and commercial lager strain (Students t-test, * $p < 0.05$, ** $p < 0.01$, *** $p < 0.001$).
