# Supplementary material for: Wild Patagonian yeast improve the evolutionary potential of novel interspecific hybrid strains for lager brewing
Source: PLoS Genet. 2024 Jun 20;20(6):e1011154. doi: 10.1371/journal.pgen.1011154 (PMC11189258; doi:10.1371/journal.pgen.1011154)
Supplement: S8 Fig — Allele frequencies over time in H3-4 and H4-1 lines evolved in T medium. In red and blue are highlighted SNPs in the genes present in the evolved individuals in the S. cerevisiae and S. eubayanus subgenome, respectively. (PDF) [file pgen.1011154.s008.pdf]

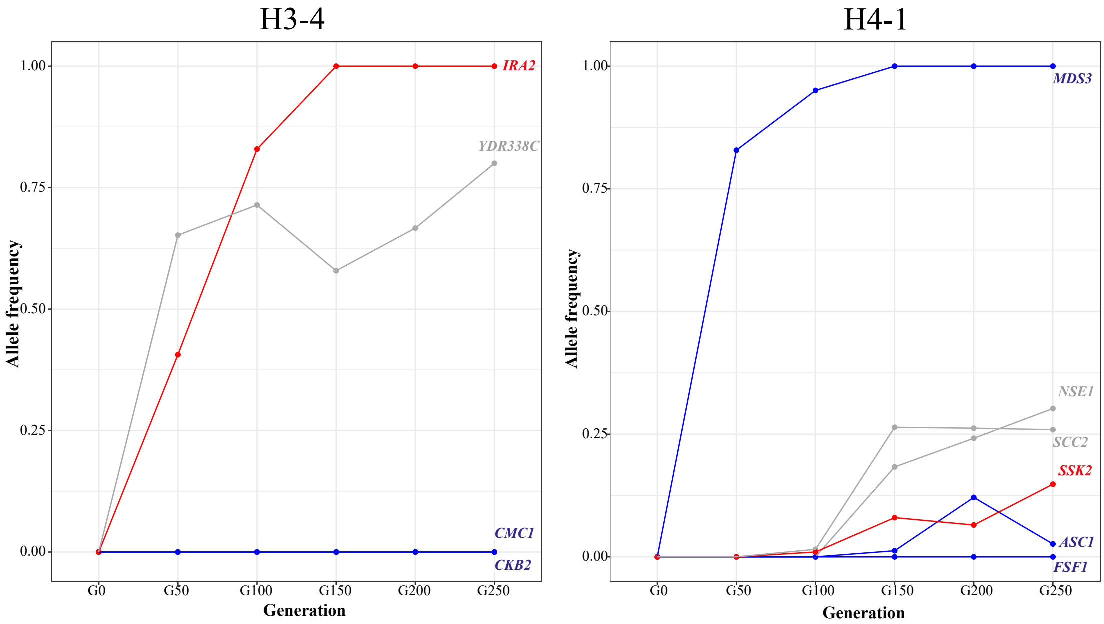

Figure S8. Dynamics of molecular evolution. Allele frequencies over time in H3-4 and H4-1line evolved in T medium. In red and blue are highlighted SNPs in the genes present in the evolved individuals in the *S. cerevisiae* and *S. eubayanus* subgenomes, respectively.
