# Supplementary material for: Wild Patagonian yeast improve the evolutionary potential of novel interspecific hybrid strains for lager brewing
Source: PLoS Genet. 2024 Jun 20;20(6):e1011154. doi: 10.1371/journal.pgen.1011154 (PMC11189258; doi:10.1371/journal.pgen.1011154)
Supplement: S9 Fig — The number of cells versus propidium iodide intensity is shown. Haploid (n), diploid (2n), and tetraploid (4n). (PDF) [file pgen.1011154.s009.pdf]

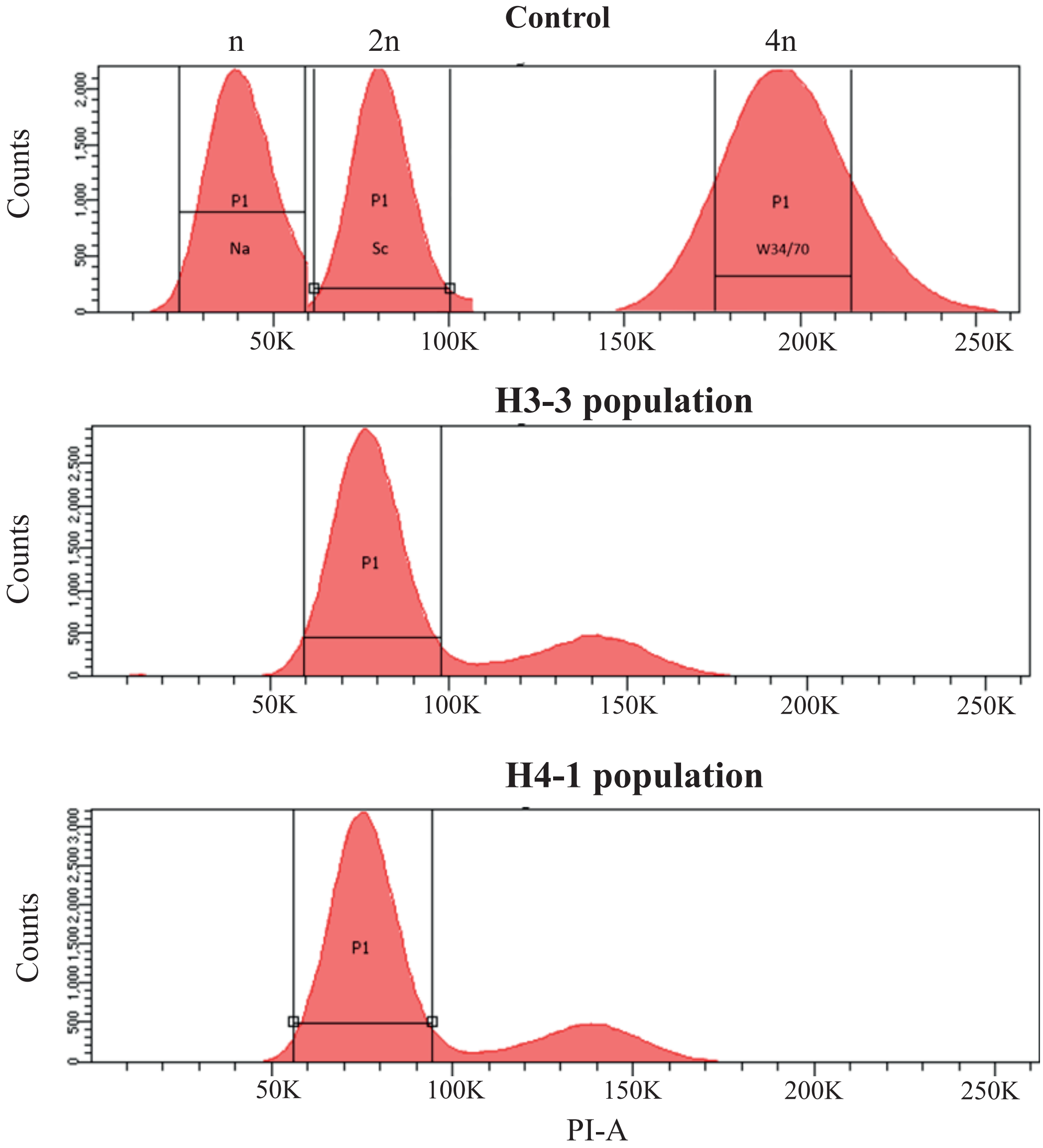

Figure S9. FACS analysis of H3-3 and H4-1 populations after 250 generations. The number of cells versus propidium iodide intensity is shown. Haploid (n), diploid (2n) and tetraploid (4n).
