# Supplementary material for: Wild Patagonian yeast improve the evolutionary potential of novel interspecific hybrid strains for lager brewing
Source: PLoS Genet. 2024 Jun 20;20(6):e1011154. doi: 10.1371/journal.pgen.1011154 (PMC11189258; doi:10.1371/journal.pgen.1011154)
Supplement: Attachment — Submitted filename: 240326_Response_to_Reviewers.pdf [file pgen.1011154.s020.pdf]

Dear Editors,

We want to thank the editors and reviewers for their time and effort in reviewing our manuscript, "*Wild Patagonian yeast improves the evolutionary potential of novel interspecific hybrid strains for lager brewing*." We have now addressed all editorial and reviewers' comments and made the suggested changes to improve our manuscript.

You will find a point-by-point response to the questions raised in the response to the reviewer's file.

Sincerely,

Fco. Cubillos

Francisco A. Cubillos
Associate Researcher iBio
Chemistry & Biology Faculty
Universidad de Santiago de Chile

UNIVERSIDAD DE SANTIAGO DE CHILE
FACULTAD DE QUÍMICA Y BIOLOGÍA
ALAMEDA 3363
Estación Central · Santiago · Chile
56-2-7181084
francisco.cubillos.r@usach.cl

Editor:

The manuscript was fully evaluated at the editorial level and by independent peer reviewers. The reviewers, especially reviewer #1, raised some important questions and concerns about the current manuscript. Based on the reviews, we will not be able to accept this version of the manuscript, but we would be willing to review a much-revised version. We cannot, of course, promise publication at that time.

Moreover, in line 59 you write "An iconic example is the domestication of the hybrid yeast Saccharomyces pastorianus". I wonder what you exactly mean by domestication here as the hybrid is itself the domesticate and therefore in the case of S. pastorianus, domestication corresponds to hybridization.

R: We agree with the Editor that this was confusing. With domestication, we mean the process through which the modern lager hybrid strain evolved. The original Bavarian hybrid was exposed to a long-term domestication process to generate the current commercial strains. We have modified the text, which now reads:

"An iconic example is the domesticated hybrid yeast Saccharomyces pastorianus".

Reviewer #1:

Molinet et al seek to generate novel S. cerevisiae x S. eubayanus hybrids to increase genetic and phenotypic diversity of lager brewing strains. They leverage natural genetic diversity of S. eubayanus strains from Chile, cross to S. cerevisiae wine strains, and phenotype hybrids for a variety of fermentation related traits. They select three hybrids to evolve under maltose + ethanol and maltose/maltortriose + ethanol conditions, evolve for 250 generations, and phenotype evolved hybrid populations and clones. They sequence the genomes of two clones, and identify a number of point mutations and copy number changes associated with sugar consumption. They find S. eubayanus mitochondria to be important in certain fermentation traits beyond the previously recognized effect on temperature. They use RNA sequencing and find increased expression of maltose genes in evolved lines and also demonstrate a role for IRA2 mutations in increasing fermentation capacity. A number of unique insights result from this work, particularly highlighting mechanisms involved in sugar sensing and catabolism.

While I think the motivation behind this study to explore greater genetic and phenotypic

diversity in lager hybrids through novel hybridization is exciting and interesting, I feel there are some additional analyses and discussion that could be addressed to strengthen the manuscript.

R: First of all, we would like to thank the reviewer for the helpful and constructive comments on our manuscript. We have now addressed all comments and made the suggested changes.

First, it was unclear to me why the authors did not sequence additional clones or populations from the evolved lines. While the two clones they did sequence revealed important genetic changes that may underlie phenotypes of interest, it is difficult to assess generalizable findings. If a goal of this paper is to understand the genetic changes underlying increased fermentation capacity, by exploring the mutations that occurred across different replicates and genotypes, the authors would have more power to conclude what changes are necessary/sufficient and repeatable.

R: We thank the reviewer for pointing this out. To describe their mutational landscape, we provide genomic data for two lines (at high coverage). We have re-examined our SNP dataset and found that many variants were already present in the parental strain or the ancestral hybrid, which we have now filtered out. As a result, at the end of the evolution experiment, we identified 1 and 2 additional variants in the H3 and H4 evolved lines, respectively (besides the mutations in IRA2 and MAL genes), which were found to be new in the evolved lines (i.e. not present in parents or the ancestral hybrid). For example, in the H4-evolved lines, we identified mutations within the SCC2 and NSE1 coding regions of two genes related to DNA replication and repair processes. Importantly, the IRA2 mutation was completely fixed in the evolved lines. The two evolved clones that we sequenced also contained the IRA2 mutation and the MAL genes duplications that our manuscript is focused on. We agree that sequencing additional clones and lines could have revealed other mutations of interest, but our budget did not allow this. Instead, we focused on whole-population sequencing of evolved H3 and H4 to provide their entire mutational landscape. Single clones may only provide a snapshop of the mutations in these populations. We now present this information more clearly in the manuscript in results and discussion sections. For example (lines 557-543):

'To detect additional genetic changes not identified in the individual clones and to track the relative frequencies of the de novo mutations in the evolution lines, we sequenced whole population samples at increasing time points of experimental evolution (at 50, 100, 150, 200, and 250 generations; Figure S8, Table S8D-E). In this way, we identified 1 and 2 additional variants in the H3 and H4 evolved lines, respectively. For example, in the H4-evolved lines, we identified mutations within the SCC2 and NSE1 coding regions of two genes related to DNA replication and repair processes (Table S8D). '

UNIVERSIDAD DE SANTIAGO DE CHILE
FACULTAD DE QUÍMICA Y BIOLOGÍA
ALAMEDA 3363
Estación Central · Santiago · Chile
56-2-7181084
francisco.cubillos.r@usach.cl

Second, since one of the stated motivations for this study is to better understand lager domestication/evolution, and since one could frame this study as a sort of lager domestication experiment, it has great potential to better understand the changes that lead to modern day lager strains. What are commonalities or differences between lager strains and evolved hybrids from this study? I understand no aneuploidy events were identified in the selected evolved hybrids (but see comment above about only sequencing two clones), but did the authors look for ploidy changes or loss of heterozygosity events?

R: We agree on the importance of identifying the differences and similarities between current commercial lager strains and our evolved hybrids. As the reviewer indicates, ploidy is one difference between lager strains and our evolved hybrids. While commercial strains have ploidy levels of or above 3n, our evolved hybrids are all diploids. We have now confirmed this with additional bioinformatic analyses (using sppIDer, a pipeline for the analysis of interspecific hybrid genomes), and flow cytometry of H3 and H4 evolved hybrids, together with their evolution lines, which in all cases confirmed pure diploidy. Both the sppIDer results and the results of flow cytometry are now highlighted in the text and were added as supplementary tables:

sppIDer results (lines 507-509):

'Genome sequencing revealed that these two strain backgrounds had equal contributions from both parental genomes, and that they had euploid, diploid genomes with no detectable aneuploidies (Table S8B)".

Flow cytometry Results: in the text (lines 545-548) and Supplementary Figure S9.

'We also determined the ploidy of this population at the end of the evolution process (250 generations), confirming that ploidy levels did not change during the experimental evolution process in the H3 population, maintaining a diploid state (Figure S9).'

In addition, we have now added an analysis of loss of heterozygosity (LOH) events in evolved hybrids. We only detected small LOH regions associated with the hybridization process itself, but no changes occurred during experimental evolution. We have included these results in the text and Table S8C. This results section now reads (lines 509-513):

'We only detected small LOH regions that were associated with the hybridization process, mainly losses of S. eubayanus DNA at subtelomeric regions (Table S8C) but also a small LOH region partly spanning the HKR1 gene located in chromosome II, between the H4A hybrid compared with the CL216.1 parent (Table S8C).'

UNIVERSIDAD DE SANTIAGO DE CHILE
FACULTAD DE QUÍMICA Y BIOLOGÍA
ALAMEDA 3363
Estación Central · Santiago · Chile
56-2-7181084
francisco.cubillos.r@usach.cl

Overall, we conclude that no aneuploidies were detected, suggesting that these may not be essential for an efficient fermentation process. We now address this in the discussion (lines 627-634):

'Interestingly, no polyploidies, aneuploidies, or loss of heterozygosity events were detected in our evolved hybrids, suggesting that these structural chromosomal changes might not be essential for efficient fermentation performance. Instead, we show that desirable phenotypic outcomes such as high ethanol production and new aroma profiles are the result of an intricate interplay of pre-existing genetic diversity, and selection on species-specific mitochondria, de novo mutations in sugar consumption genes, together with CNV of the MAL genes, important to improve maltose consumption during fermentation."

Are any of the identified mutations present in previously sequenced beer strains? This would be really interesting to discuss further, as the lager strains have quite complex karyotypes, and if the authors can recapitulate a lot of lager fermentation traits without ploidy, aneuploidy, and LOH, this could suggest that many of those changes are not necessary for lager fermentation (although the cold phenotype is not assessed here, so that context may be less clear).

Thank you for this suggestion. Variation in chromosomal location and copy number of the MAL genes are indeed present in many industrial lager strains, typically containing six or more copies of the MAL3 locus (Goncalves et al., 2016). Furthermore, all currently used lager strains have the S. eubayanus mitochondria consistent with our study, which revealed a larger improvement in fermentation capacity in hybrids carrying S. eubayanus mitochondria. Although we did not identify any other similarities between our evolved hybrids and industrial strains, it is important to highlight that current commercial strains went through a population bottleneck created by the isolation and propagation of pure cultures during the industrialization of brewer's yeast. Therefore, the genetic pathways we identified are not necessarily the only possible route to increase fermentation capacity of lager strains. For that reason, in the results and discussion sections, we highlight mutations related to carbon metabolism, providing direct evidence of the link between carbon uptake and fermentation capacity. We now discuss this in the manuscript (lines 698-703):

'We further consolidated this mechanism by CNV and transcriptome analyses, which detected several up-regulated genes related to maltose consumption in the evolved hybrid during fermentation. Fluctuations in chromosomal location and copy number of the MAL genes are present in many industrial strains [85] and de novo evolved hybrids [29], containing six or more copies of the MAL3 locus.'

In addition, we now added a comparison of the mutations found in our hybrids with previous studies. E.g., Krogerus et al. (2018) found missense and frameshift mutation in

the S. cerevisiae IRA2 allele in two evolved strains and copy number variations in MAL31 and MALL11. Gorter de Vries et al. (2019) also found mutations in the IRA2 gene in multiple evolved isolates. To highlight this, we have added a paragraph in the discussion addressing the reviewer's recommendation (lines 677-682):

'Evolved hybrids carried a premature stop codon in the IRA2 gene, absent in both the S. cerevisiae and S. eubayanus parental ancestors, and in the unevolved hybrids at the beginning of experimental evolution. Previous studies, despite using different experimental conditions and hybrid genetic backgrounds, have also identified mutations in the IRA2 gene in multiple evolved isolates, corroborating the important role of this gene in the fermentation process.'

Third, I might suggest a stylistic choice to condense some of the phenotypic results presented in Figures 1 and 2, and the associated text, in order to highlight the insights from the evolution experiments and the genomic, genetic, and transcriptomic results, which are quite interesting and feel a little de-emphasized in the manuscript.

R: We have now reduced the length of the phenotypic results to emphasize our genetic and transcriptomic findings.

Minor comments

Can the authors comment on their S. cerevisiae strain selection choice? Did they consider using a beer strain?

R: Previous studies have used beer strains to generate interspecific hybrids (Krogerus et al., 2018; Gorter De Vries et al., 2019). For our study, we wanted to use only strains isolated in Chile, of both species. In particular, the Chilean S. cerevisiae strains were collected from areas related to wine production. For the sake of brevity, we have not included this in the results section. However, the methods section provides detailed information on the strains' geographic and genetic origins.

Several of the figures were a bit challenging for me to understand. For example in Figures 2-4, I think clarity could be improved by including more text on the figures and in the figure legend to demonstrate which population is being compared to what (statistics, relative growth compared to other evolved lines or to the ancestor, etc). For Figure 2 - Is the only theorized difference between the hybridizations at 12 or 20 which species mtDNA they inherit? If so, I suggest changing the figure legend from hybridization temperature to S. cerevisiae mtDNA and S. eubayanus mtDNA.

R: As suggested, we have modified the figure legends adding more information about statistical methods, and included additional information in the different plots about

UNIVERSIDAD DE SANTIAGO DE CHILE
FACULTAD DE QUÍMICA Y BIOLOGÍA
ALAMEDA 3363
Estación Central · Santiago · Chile
56-2-7181084
francisco.cubillos.r@usach.cl

fitness values relative to the ancestral hybrids. We changed "temperature hybridization" to "S. cerevisiae/eubayanus mitochondria".

Please provide additional information and context for the volatile analysis. Are the compounds identified in the novel hybrids pleasant or aversive? Are there changes in volatiles between ancestor and evolved hybrids? The authors note the presence of 4VG, which is unfavorable in many styles of beer including lager. The authors mention several times that one of the motivations for new lager hybrids is to generate novel aromas/flavors, but it is unclear if the compounds found here would be desirable or not, and what the authors think would be needed to address this.

R: Thank you for these suggestions. We focused on the differences in volatile compound production with distinct aroma effects. For example, the evolved strain produces 1-octanol and 4-vinylphenol, while the commercial strain does not. These could provide sweetish notes for 1-octanol and clove-like, spicy, and phenolic aromas in the case of 4-vinylphenol. The latter is commonly found in Belgian Ale beers and wheat beers. In contrast, the commercial strain produces citronellol, which is associated with a citrus aroma. Therefore, the beers produced with the evolved strain would have a different profile, towards a more herbal, spicy, and phenolic character. In contrast, those produced with the commercial strain would have a more citrusy and refreshing profile. Differences were the main aim of our work, rather than judging whether herbal/spicy profiles are preferred over citrusy, which the consumer decides. We have now added this description to the manuscript (lines 497-501):

'These results demonstrate that the aroma profiles of the evolved hybrid differ from the commercial lager strain. Therefore, the beers produced with the evolved hybrid would have a different profile towards a more herbal, spicy, and phenolic character. In contrast, those produced with the commercial strain would have a more citrusy and refreshing profile.'

Can the authors comment on the number of SNPs identified between the two hybrids? Do they suspect a mutator allele in the one evolved line?

R: Thank you for this interesting thought. The number of SNPs in the two evolved hybrids was determined by comparing evolved versus ancestral strains. We did not compare the two evolved hybrids directly because they do not share the same ancestral background. However, we can compare if both strains share genes or pathways with de novo SNPs, and we did not find any shared genes with de novo SNPs.

We do not suspect a mutator allele for two reasons. First, both evolved lines (H3-4 and H4-1) exhibit a similar number of SNPs (2 and 4 SNPS for H3 and H4, respectively. Information now added to Table S8E). Second, the final number of de novo SNPs is low (mutation rate of ~$8.3x10^{-11}$ per haploid genome and generation) compared to mutation

rates in YPD at 30 °C, which is about $6.3x10^{-5}$ per haploid genome and generation (Joseph and Hall, 2004). If a a mutator allele was present, we would expect a higher mutation rate.

A little more explanation about the ira2 knockout creation/results would be helpful. Is the ira2 knockout in H3E just a total deletion of the ira2 gene in the evolved hybrid background that already has a IRA2 premature stop codon? Since the premature stop has a different phenotype than the null, the authors should be careful with the language about this result, since they did not test the effect of the premature stop codon. This finding about complex interactions is very interesting. Since this mutation occurred early in the experiment, were there any other de novo mutations in this background that could explain this interaction?

R: The ira2 knockout in H3-E is a total deletion of the S. cerevisiae IRA2 allele that already has the premature stop codon. Unfortunately, we could not generate the IRA2 allele version containing an early stop codon in the ancestral strain, or clone the ancestral allele into the evolved hybrid. We aimed to be careful with our language, indicating that likely genetic interactions underlie the IRA2 allele effect.

We have now also checked SGD for genetic interactions between IRA2 and genes with de novo SNPs, CNVs or DEGs. We found putative genetic interactions between IRA2 with IMA1, CMC1, and MAL11. CMC1 is involved in the assembly of cytochrome c oxidase in the mitochondria and contained a SNP in the corresponding S. eubayanus parental allele. IMA1 and MAL11 were up-regulated and exhibited CNVs in the evolved hybrid. Interactions between the S. cerevisiae IRA2 allele with these genes could explain the differences observed between the IRA2 null mutant and the hybrid with the premature stop codon. We have now included information about this in the discussion following the reviewer's suggestion (lines 682 – 692):

'Null $ira2^{SC}$ mutants did not show the same phenotypes as the H3-E hybrid with the IRA2 premature stop codon, suggesting a complex genetic interaction in this genetic background. We searched for genes with de novo mutations previously described to have genetic interactions with IRA2 using the Saccharomyces Genome Database (SGD). Our search resulted in three genes potentially interacting with IRA2: IMA1, MAL11, and CMC1. CMC1 encodes for a protein involved in the assembly of cytochrome c oxidase in the mitochondria [81] and contains a SNP in the corresponding S. eubayanus parental allele. In addition, IMA1 and MAL11 genes were up-regulated in the evolved hybrid and exhibited CNVs relative to the ancestral hybrid. Interactions between the S. cerevisiae IRA2 allele with these genes could explain the differences observed between the $ira2^{SC}$ mutants and the H3-E hybrid.'

Reviewer #2:

Summary: Molinet et al. is one of the most comprehensive experimental evolution studies I have read. To summarize, the authors first hybridized three Saccharomyces cerevisiae and Saccharomyces eubayanus strains at two temperatures (12 and 20C) to select for specific parental mitochondria. These hybrids were phenotypically assessed relative to the parental strains and no significant differences were found. The authors then experimentally evolved hybrids under two conditions maltose (M) and maltose/maltotriose (T) conditions for 250 generations (saving generations along the way). These evolved strains were then phenotypically, genomically, transcriptomically, and metabolically assessed for variation between hybrids, ancestral, and commercial strains. The authors found multiple differences in hybrids relative to each other, the ancestral strains, and the commercial strains across the multiple aspects they tested. Overall, they found hybridization combined with experimental evolution/selection can result in yeasts with relatively enhanced fermentation capacities.

Major Concerns:

I have no major concerns for this manuscript. It was well written, comprehensive, the experimental design was good, and all analyses were sound and reasonable.

R: We would like to thank the reviewer for the comments and the encouraging feedback on our work. We have now addressed all of the suggestions and incorporated the suggested changes in the manuscript.

Minor Concerns:

Line 28 there is an extra space between We and used.

R: This has been corrected.

In figure 2E for the Evolved Lines T, is there no boxplot because most are at 100% uptake? For 2F, the additional colors for the boxplots should either be included in the legend (similar to figure S7), or the colors should be removed.

R: It is correct that in Figure 2E for the T evolved lines, there is no boxplot because most strains showed 100% maltotriose uptake. Figure 2F has now been corrected.

For the volatile compounds section (Lines 481 – 492) could you elaborate on the impact of the differences in volatile compounds between the commercial strain and the evolved strains?

UNIVERSIDAD DE SANTIAGO DE CHILE
FACULTAD DE QUÍMICA Y BIOLOGÍA
ALAMEDA 3363
Estación Central · Santiago · Chile
56-2-7181084
francisco.cubillos.r@usach.cl

R: This recommendation was also suggested by Reviewer #1. Here is our answer to this comment:

R: Thank you for these suggestions. We focused on the differences in volatile compound production with distinct aroma effects. For example, the evolved strain produces 1-octanol and 4-vinylphenol, while the commercial strain does not. These could provide sweetish notes for 1-octanol and clove-like, spicy, and phenolic aromas in the case of 4-vinylphenol. The latter is commonly found in Belgian Ale beers and wheat beers. In contrast, the commercial strain produces citronellol, which is associated with a citrus aroma. Therefore, the beers produced with the evolved strain would have a different profile, towards a more herbal, spicy, and phenolic character. In contrast, those produced with the commercial strain would have a more citrusy and refreshing profile. Differences were the main aim of our work, rather than judging whether herbal/spicy profiles are preferred over citrusy, which the consumer decides. We have now added this description to the manuscript (lines 497 – 501) :

'These results demonstrate that the aroma profiles of the evolved hybrid differ from the commercial lager strain. Therefore, the beers produced with the evolved hybrid would have a different profile towards a more herbal, spicy, and phenolic character. In contrast, those produced with the commercial strain would have a more citrusy and refreshing profile.'

Reviewer #3:

The manuscript by Molinet et al. describes the generation of new S. cerevisiae x S. eubayanus hybrids and their improvement through experimental evolution. The evolved hybrids outperform the ancestral hybrids and the parental strains in wort fermentations. Whole genome sequencing and RNAseq are used to identify genetic changes in the evolved hybrids, which might explain the enhanced fermentation. The study is technically sound, and the manuscript is well written and is a useful contribution to the yeast and brewing community. I only have a couple of comments and suggestions for improving it further:

R: We would like to thank the reviewer for the helpful comments on our manuscript. We have now addressed all of the comments and made the suggested changes.

General comment: you use experimental evolution on the hybrids to improve the fermentation capacity beyond the parental strains. Isn't it likely that one could have seen a similar improvement by performing the experimental evolution on the parental strains directly (e.g. we saw this in dx.doi.org/10.1128/AEM.02302-17)? You could

maybe comment on this in the discussion, and also explain what the potential benefits of including the hybridization are.

R: Indeed, improving the parental strains' fitness is an alternative strategy towards the same final objective. This has been demonstrated in Krogerus et al. (2018), for S. cerevisiae and by Mardones et al. (2021) for S. eubayanus. However, one of the main aims of our work was to increase the genetic diversity and the phenotypic profile of de-novo lager hybrid strains by combining different subgenomes. In the case of S. eubayanus, the main problem with this species is its poor maltotriose consumption, a trait not easily improvable through artificial selection compared to commercial hybrid strains. On the other hand, our study shows that carrying mitochondria from S. eubayanus provides a selective advantage under fermentation conditions and leads to greater evolutionary potential in hybrids. Thus, the hybrids combine traits of both parents, such as low-temperature tolerance and maltotriose uptake. In addition, the gene expression subgenomes crosstalk may generate different volatile compound combinations, increasing the variety of flavors and aromas. Finally, experimental evolution with hybrid strains also provides an opportunity to study other fundamental evolutionary processes, such as the genomic stabilization process of hybrid genomes over time.

We have now added this to the discussion (lines 651 – 659) :

'An alternative is to obtain improved strains by performing experimental evolution on parental strains, as previously described [44,78]. However, S. eubayanus has poor maltotriose consumption, a trait not easily improvable through artificial selection compared to commercial strains [79]. Instead, hybridization has the potential to produce more genetic variation than mutations alone. Thus, the hybrids combine traits of both parents, such as low-temperature tolerance and maltotriose uptake. In addition, the gene expression subgenomes crosstalk may generate different volatile compound combinations, increasing the variety of flavors and aromas [34].'

Throughout. 'Lager' -> 'lager'

R: This has been corrected.

Line 38. Greater

R: This has been corrected.

Lines 125-127. What is the genetic background of these strains? Are any of them brewing strains (Ale beer or Mosaic beer) or isolated from brewing environments?

R: All S. cerevisiae strains were isolated from different wine-producing areas in Central Chile. Unfortunately, they are not sequenced, but strains from the same area and similar origin were sequenced in the work of Peter et al. (2018) (DOI: 10.1038/s41586-018-0030-5), and all these strains were assigned to the Wine/European clade. This information is available in Table S1.

Line 332. Maybe "such as growth on glucose, maltose, maltotriose, and in the presence of ethanol or sorbitol"?

R: This has been corrected and now reads: "such as glucose, maltose, maltotriose, in the presence of ethanol and simulating osmotic stress with sorbitol".

Figure 1. Could you clarify what the parameters 'Diauxic shift (lag time)' and 'Diauxic shift (umax)' are?

R: We evaluated the hybrid strains under carbon source switching (diauxic shift) from glucose to maltose. This is described in the methodology section (subsection phenotypic characterization). Strains were first grown in glucose, and then transferred to fresh medium containing maltose as the sole carbon source, where OD was measured. Lag time represents the initial period in the cell population when cells adjust to the second carbon source (maltose) before starting exponential growth (Diauxic umax). To clarify, we now included a description of these parameters in the Figure 1 caption and the Methods section (lines 168 – 171).

Figure 3A and B. Shouldn't mass loss as CO_2 and ethanol formation be strongly correlated? Here the strain with the highest ethanol formation didn't have the highest CO_2 produced. Maybe worth double-checking the numbers.

R: Thank you for this suggestion. We have double-checked the numbers to make sure they are correct. While a direct stoichiometric relationship exists between sugar consumption, ethanol production, and CO_2 loss, ethanol is not the only byproduct of alcoholic fermentation. Cells can also produce glycerol, acetic acid, and lactic acid, impacting ethanol yield among strains, which may redirect metabolic fluxes towards producing these compounds. Unfortunately, we did not measure the production of these other compounds to correlate them with CO_2 production, and the observed differences with ethanol production. We have added this comment in the discussion section (line 648 - 651):

"We also identified an evolved individual that produces significantly more ethanol than the commercial lager strain, likely due to differences in metabolic fluxes between them. For example in the production of glycerol, acetic acid, and lactic acid, as well as in the consumption of more complex sugar sources".

UNIVERSIDAD DE SANTIAGO DE CHILE
FACULTAD DE QUÍMICA Y BIOLOGÍA
ALAMEDA 3363
Estación Central · Santiago · Chile
56-2-7181084
francisco.cubillos.r@usach.cl

Line 468. 3.6 - 3.8% ABV is quite low for a 12P wort. Was there a lot of unfermented sugars still in the beers?

R: We measured glucose, fructose, maltose, and maltotriose before and after fermentation. All evaluated evolved individuals had 100% sugar consumption, except for the ancestral hybrids, which did not consume maltotriose. In contrast, the commercial strain reached levels above 4% v/v. The lower alcohol content in some evolved hybrids may be due to other sugars present in the wort that were not measured and/or consumed, such as sucrose, and the production of other byproducts such as glycerol, acetic acid, and lactic acid, as described earlier. We have now included this in the discussion section as mentioned in the previous comment.

Figure 3C and Lines 484-491. In the heatmap labels you have fatty acids (e.g. hexanoic acid), but in the text you talk about ethyl esters of these fatty acids. Octanoic acid is also present twice in the heatmap. In the heatmap, is 3-methylbutyl ester 3-methylbutyl acetate? In the text you use 4-vinyl guaiacol, while in the heatmap you have 2-methoxy-4-vinylphenol.

R: Thank you, the labels in Figure 3C have been corrected. They now show the full name of each compound. In this case, hexanoic acid corresponds to hexanoic acid, ethyl ester, or ethyl hexadecanoate, a long-chain fatty acid ethyl ester. We have modified the text specifying that these compounds correspond to fatty acid ethyl esters. We also specified the different octanoic acids (octanoic acid ethyl ester, octanoic acid 3-methylbutyl ester or 3-methylbutyl octanoate, and octanoic acid). In the case of 3-methylbutyl ester, it corresponds to octanoic acid 3-methylbutyl ester or 3-methylbutyl octanoate. We have modified 4-vinyl guaiacol to 4-vinylphenol in the text.

Line 531 instead of "CRISPR assay", maybe just "CRISPR-Cas9 gene editing"?

R: Thank you, we have made the suggested change.

UNIVERSIDAD DE SANTIAGO DE CHILE
FACULTAD DE QUÍMICA Y BIOLOGÍA
ALAMEDA 3363
Estación Central · Santiago · Chile
56-2-7181084
francisco.cubillos.r@usach.cl
