# Supplementary material for: Wild Patagonian yeast improve the evolutionary potential of novel interspecific hybrid strains for lager brewing
Source: PLoS Genet. 2024 Jun 20;20(6):e1011154. doi: 10.1371/journal.pgen.1011154 (PMC11189258; doi:10.1371/journal.pgen.1011154)
Supplement: Attachment — Submitted filename: Summary_PGenetics.docx [file pgen.1011154.s019.docx]

Summary: Molinet *et al*. is one of the most comprehensive experimental evolution studies I have read. To summarize, the authors first hybridized three *Saccharomyces cerevisiae* and *Saccharomyces eubayanus* strains at two temperatures (12 and 20C) to select for specific parental mitochondria. These hybrids were phenotypically assessed relative to the parental strains and no significant differences were found. The authors then experimentally evolved hybrids under two conditions maltose (M) and maltose/maltotriose (T) conditions for 250 generations (saving generations along the way). These evolved strains were then phenotypically, genomically, transcriptomically, and metabolically assessed for variation between hybrids, ancestral, and commercial strains. The authors found multiple differences in hybrids relative to each other, the ancestral strains, and the commercial strains across the multiple aspects they tested. Overall, they found hybridization combined with experimental evolution/selection can result in yeasts with relatively enhanced fermentation capacities.

**Major Concerns:**

I have no major concerns for this manuscript. It was well written, comprehensive, the experimental design was good, and all analyses were sound and reasonable.

**Minor Concerns:**

Line 28 there is an extra space between *We* and *used*

In figure 2E for the Evolved Lines T, is there no boxplot because most are at 100% uptake? For 2F, the additional colors for the boxplots should either be included in the legend (similar to figure S7), or the colors should be removed.

For the volatile compounds section (Lines 481 – 492) could you elaborate on the impact of the differences in volatile compounds between the commercial strain and the evolved strains?
