# Supplementary material for: Wild Patagonian yeast improve the evolutionary potential of novel interspecific hybrid strains for lager brewing
Source: PLoS Genet. 2024 Jun 20;20(6):e1011154. doi: 10.1371/journal.pgen.1011154 (PMC11189258; doi:10.1371/journal.pgen.1011154)
Supplement: Attachment — Submitted filename: Pgenetics_R2.docx [file pgen.1011154.s021.docx]

**Summary**: I previously reviewed the Molinet *et al*. manuscript and thought it was a well written comprehensive study. In my previous review I thought there were only minor issues and should be accepted. I feel that the authors have sufficiently addressed my comments and appreciate that they took the time to elaborate on the differences in volatile compounds produced by the evolved strains versus the commercial strains. Overall, I think the manuscript is even stronger than it was before and should be accepted.
